# Effectiveness of a School Physician-Led Counseling Intervention on Cholesterol Levels and Lifestyle Behaviors in Children with Hypercholesterolemia: A Randomized Controlled Trial

**DOI:** 10.3390/children13070848

**Published:** 2026-06-24

**Authors:** Katarina Tomelić Ercegović, Josipa Glavaš, Ivana Sikirica, Andrea Vrdoljak, Helena Tokić, Jelica Perasović, Željka Karin

**Affiliations:** Department of School and Adolescent Medicine, Teaching Institute for Public Health, Split-Dalmatia County, 21000 Split, Croatiazeljka.karin@nzjz-split.hr (Ž.K.)

**Keywords:** children, hypercholesterolemia, intervention, Mediterranean diet, physical activity, prevention

## Abstract

**Highlights:**

**What are the main findings?**

- The physician-led counseling intervention showed a favorable but non-significant trend toward lower total cholesterol (TC) levels in children with elevated cholesterol levels and a negative family history of familial hypercholesterolemia (FH).
- The intervention was associated with improved adherence to the Mediterranean diet and favorable exploratory changes in selected physical activity and sedentary behavior outcomes.

**What are the implications of the main findings?**

- Physician-led counseling within school healthcare settings may help support healthier lifestyle awareness and selected health-related behaviors among children and families.
- Further studies with larger samples, longer follow-up periods, and comprehensive lipid assessment are needed to confirm the effectiveness of this approach, including in children with elevated cholesterol levels and a positive family history of FH.

**Abstract:**

**Background**: This randomized controlled trial aimed to evaluate the effects of a school physician-led counseling intervention on total cholesterol (TC) levels, adherence to the Mediterranean diet, physical activity, and sedentary behavior in children aged 6–7 years with elevated cholesterol levels in a Mediterranean setting. **Methods**: A one-year randomized controlled study was conducted among children aged 6–7 years with elevated TC levels, excluding those with familial hypercholesterolemia (FH). Participants were randomly assigned to either a control group (*n* = 38) or an intervention group (*n* = 39). All participants received standard care consisting of educational materials and baseline counseling, while the intervention group additionally participated in three structured follow-up counseling sessions conducted by school physicians during the one-year study period. Counseling focused on Mediterranean dietary habits, implementation of basic dietary principles in cases of elevated TC levels, promotion of physical activity, and reduction in sedentary behavior. TC levels were measured at baseline and at the end of the study. Dietary habits, physical activity, and sedentary behavior were assessed using validated questionnaires. For the primary outcome, a descriptive change-from-baseline analysis, unadjusted mean difference, the approximate 95% confidence interval, and Cohen’s d effect size were calculated. **Results**: At baseline, no significant differences in TC levels were observed between groups (*p* = 0.852). After the intervention, mean TC levels were lower in the intervention group than in the control group (4.977 ± 0.414 mmol/L vs. 5.137 ± 0.410 mmol/L); however, the between-group difference did not reach statistical significance (*p* = 0.089). The unadjusted mean difference at follow-up was −0.160 mmol/L, with an approximate 95% confidence interval from −0.35 to 0.03 and a small-to-moderate effect size in favor of the intervention group (Cohen’s d = −0.39). Descriptive change-from-baseline analysis showed a greater mean reduction in TC in the intervention group than in the control group (−0.364 mmol/L vs. −0.195 mmol/L). A statistically significant improvement in adherence to the Mediterranean diet was observed in the intervention group compared with the control group (*p* < 0.001). Favorable changes were also observed in several physical activity and sedentary behavior variables, including participation in organized physical activity, walking and running activities, and reduced television viewing and video gaming time. Given the exploratory nature of behavioral analyses and the number of physical activity and sedentary behavior outcomes examined, these findings should be interpreted cautiously. **Conclusions**: The school physician-led counseling intervention did not result in a statistically significant between-group difference in TC levels after one year, although the direction and magnitude of change favored the intervention group. The intervention was associated with improved adherence to the Mediterranean diet and favorable exploratory lifestyle-related behavioral changes. Nevertheless, the findings should be interpreted cautiously in light of the relatively small sample size, non-significant primary outcome, and exploratory nature of behavioral analyses.

## 1. Introduction

Cardiovascular diseases (CVDs) remain the leading cause of mortality worldwide and in Croatia, accounting for approximately 17.9 million deaths annually [1,2]. The development of cardiovascular risk factors often begins in childhood, where unhealthy dietary habits, reduced physical activity, sedentary behavior, obesity, and genetic predisposition contribute to the early onset of dyslipidemia and atherosclerosis [3]. Early implementation of nonpharmacological measures, including healthy dietary habits and regular physical activity, is therefore considered a key strategy in cardiovascular disease prevention [4].

Familial hypercholesterolemia (FH) is the most common inherited disorder of lipid metabolism and is characterized by markedly elevated low-density lipoprotein cholesterol (LDL-C) levels from early childhood [5]. Due to lifelong exposure to elevated cholesterol levels, individuals with FH have a substantially increased risk of premature cardiovascular disease compared with the general population [6]. The prevalence of FH is estimated at 1:200 to 1:250 individuals; however, most affected individuals remain undiagnosed [7]. In Croatia, the estimated number of individuals with FH ranges between 10,000 and 20,000 [8].

In Croatia, all children undergo mandatory systematic health examinations during their schooling, according to the National Plan and Program of Preventive Healthcare Measures for School and Adolescent Medicine in Croatia [9]. These examinations are conducted by specialists in school and adolescent medicine (school physicians) and include systematic medical examinations, health screening, vaccination programs, health education, counseling, and monitoring of physical, mental, and social health, with the aim of early disease detection, prevention, and promotion of healthy lifestyles. Because these examinations are mandatory and population-based, school physicians have valuable insight into the health status of children within their region and play an important role in preventive healthcare and early identification of cardiovascular risk factors.

**Figure 1:**
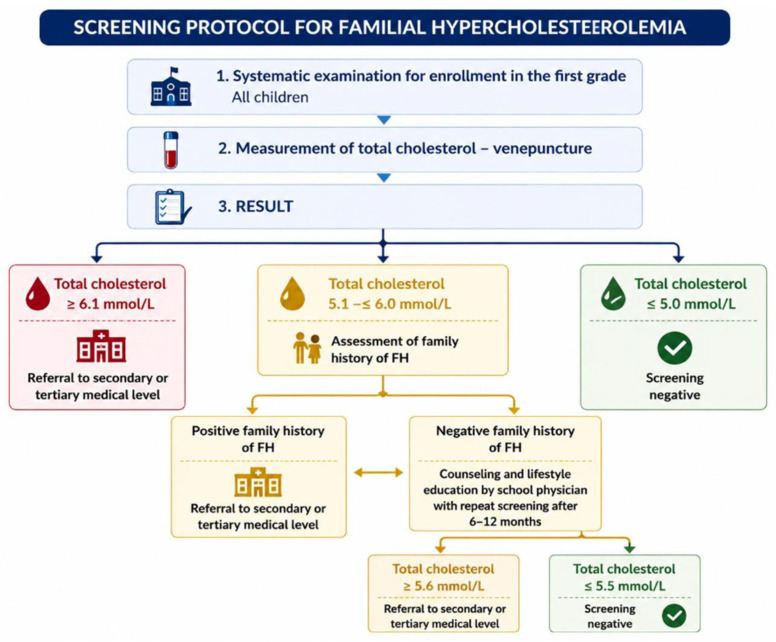
Screening protocol for familial hypercholesterolemia in children during first-grade enrollment examinations in Croatia.

In 2023, Croatia introduced the National Program for Screening and Early Detection of FH (Croatian National Program), representing a universal screening program for children aged 6–7 years. Screening is performed during the medical examination prior to enrollment in the first grade of primary school and is conducted by school physicians. The program is based on a reverse-cascade screening model in which children with elevated TC levels and a positive family history are referred for further evaluation, while children with elevated TC levels but a negative family history remain under the care of school physicians [10] [Figure 1]. This population-based screening approach creates an opportunity not only for early detection of FH but also for implementation of early lifestyle interventions in children with elevated TC levels who do not meet the criteria for FH.

Accurate data on the prevalence of dyslipidemia in children are limited because of differences in diagnostic criteria and the lack of systematic screening programs in many countries. Previous studies have reported that 6.1% of children and adolescents in Germany had LDL cholesterol levels ≥ 130 mg/dL (3.4 mmol/L) [11], while approximately 20% of children aged 6–19 years in the United States have some form of dyslipidemia [12]. In Spain, 27.9% of children were reported to have TC levels above 200 mg/dL (5.2 mmol/L) [13]. In Split-Dalmatia County (SDC), a Mediterranean region of Croatia, approximately 7% of children aged 6–7 years have elevated TC levels [14]. Universal childhood screening programs for FH are currently implemented in Slovenia, the United Kingdom, Norway, the Czech Republic, and Spain [15,16,17], while some countries, including the Netherlands and Austria, have discontinued such programs [18]. Croatia is therefore among the few countries implementing universal childhood screening for FH.

Several studies have investigated counseling interventions aimed at improving lipid profiles and lifestyle habits in children; however, important differences exist regarding study populations, intervention duration, healthcare providers, and measured outcomes. Long-term interventions conducted in Finland reported favorable effects on dietary habits and some lipid parameters among children from the general population [19,20]. Other studies also reported improvements in lipid profiles following dietary interventions in children [21]. However, to our knowledge, no previous study has specifically evaluated counseling interventions delivered by school physicians among children with elevated cholesterol levels but without confirmed familial hypercholesterolemia.

The primary aim of this study was to evaluate the effects of a school physician-led counseling intervention on TC levels among children with screening values between 5.1 and 6.0 mmol/L and a negative family history of FH. Secondary outcomes included adherence to the Mediterranean diet, physical activity, and sedentary behavior.

## 2. Materials and Methods

As part of the Croatian National Program, all children from SDC, a Mediterranean region of Croatia, who had reached the age of 6 years by 1 April 2024 underwent systematic medical examinations prior to enrollment in the first grade of primary school. TC levels were measured in authorized primary healthcare medical–biochemical laboratories.

For children with TC values between 5.1 mmol/L and 6.0 mmol/L, parents or legal guardians completed the “Questionnaire for parents of children with elevated TC detected during screening” with the assistance of school physicians in order to assess family history of FH. If all questionnaire responses were negative, the family history of FH was considered negative.

Anthropometric measurements of body height and body weight were performed by graduate nurses within school and adolescent medicine teams, while waist circumference was measured by school physicians. All parents received printed educational materials prepared by the Croatian Institute of Public Health and the Department of Nutrition and Dietetics of the University Hospital Centre Zagreb regarding dietary recommendations for children with elevated TC levels, together with World Health Organization educational materials related to physical activity recommendations for children and adolescents aged 5–17 years. In addition, all participants received baseline counseling by school physicians regarding Mediterranean dietary habits, physical activity, and healthy lifestyle behaviors.

Parents or legal guardians who agreed to participate in the study signed written informed consent forms and completed the KIDMED questionnaire (Mediterranean Diet Quality Index) and the Pre-PAQ questionnaire (Preschool-age Physical Activity Questionnaire).

A total of 87 children met the inclusion criteria and were randomized using the Research Randomizer tool into either a control group (*n* = 44) or an intervention group (*n* = 43). The random allocation sequence was generated by a researcher using Research Randomizer (Version 4.0; Urbaniak and Plous, Social Psychology Network, Middletown, CT, USA; available at randomizer.org). Eligible participants were enrolled by school physicians after completion of screening examinations and informed consent procedures. After enrollment, the same researcher assigned participants to the intervention or control group according to the generated allocation sequence. Allocation was performed after eligibility confirmation and informed consent; however, formal allocation concealment procedures were not implemented. Due to the nature of the counseling intervention, blinding of participants, parents/legal guardians, and school physicians was not possible.

**Figure 2:**
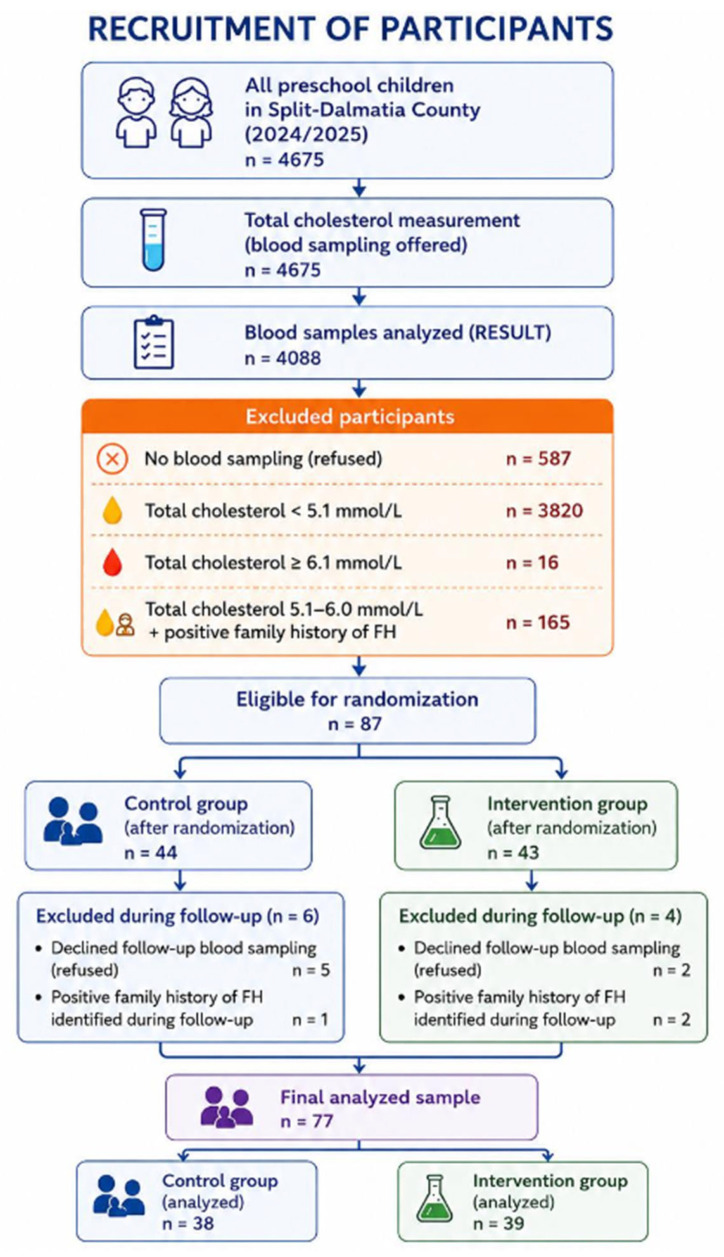
Flowchart of participant recruitment and selection in the study conducted among preschool children in Split-Dalmatia County (2024/2025). Participants were excluded based on cholesterol levels, lack of blood sampling, or family history criteria. The final sample was divided into control and intervention groups.

During follow-up, parents of seven children withdrew participation, while three children were excluded after additional family history assessment identified a positive FH history. Consequently, the final study sample consisted of 77 children, including 38 children in the control group and 39 children in the intervention group [Figure 2].

Children in the control group received standard care consisting of baseline counseling and educational materials only. One year later, they were invited to School and Adolescent Medicine Clinics in SDC for repeated TC measurement, anthropometric assessment, and completion of the KIDMED and Pre-PAQ questionnaires.

Children in the intervention group received additional structured counseling conducted by school physicians on four occasions during the one-year study period: once in person at School and Adolescent Medicine Clinics in SDC for approximately 30 min in May 2024 and three follow-up telephone counseling sessions lasting 25–30 min in September 2024, January 2025, and May 2025. Counseling sessions focused on Mediterranean dietary habits, basic dietary recommendations for children with elevated TC levels, promotion of physical activity, reduction in sedentary behavior, and implementation of healthy lifestyle habits within the family environment. During counseling sessions, school physicians discussed the content of educational materials with parents and encouraged realistic and achievable lifestyle modifications adapted to the child’s family and social environment. In June 2025, TC measurement, anthropometric measurements, and the KIDMED and Pre-PAQ questionnaires were repeated.

The counseling intervention conducted by school physicians aimed to promote healthy dietary habits and increase physical activity in children. Dietary recommendations were based on Mediterranean dietary principles as well as on basic dietary recommendations for children with elevated TC levels and were adapted according to the child’s age, body weight, height, and physical activity level. Parents were advised to reduce intake of foods associated with adverse health effects and increase consumption of foods with favorable nutritional characteristics. Counseling regarding physical activity included recommendations for age-appropriate sports participation and reduction in sedentary behaviors. Individual environmental and health-related circumstances were considered in order to establish realistic lifestyle goals.

Physical activity and sedentary behavior were assessed using the Pre-PAQ questionnaire, a validated parent-reported instrument designed to evaluate activity levels and sedentary behaviors in young children across different intensities and daily contexts. The questionnaire includes items related to walking, participation in organized physical activity, running intensity, transportation habits, television viewing, and computer or video game use. Although originally developed for preschool-aged children, the questionnaire was considered appropriate for the present study because the participants were children aged 6–7 years who were transitioning from preschool to the first grade of primary school. At this developmental stage, parent-reported assessment remains relevant, and the questionnaire captures physical activity and sedentary behaviors characteristic of this transitional age period. The Croatian version of the questionnaire demonstrated satisfactory reliability in a previous study by Karin et al. (2020) [22].

Adherence to the Mediterranean diet was assessed using the Croatian version of the KIDMED questionnaire, whose reliability was demonstrated in a previous study by Štefan et al. (2017) [23]. The KIDMED index includes 16 items: 12 positively scored items (+1) and 4 negatively scored items (−1). The raw score ranges from −4 to 12; however, following the standard interpretation of the KIDMED index, negative values are recoded as 0, yielding a final score range of 0–12. Higher scores indicate greater adherence to the Mediterranean diet. Scores ≥ 8 reflect good adherence, scores 4–7 average adherence, and scores ≤ 3 poor adherence.

The questionnaire used to assess family history of FH consisted of three questions related to elevated lipid levels or lipid-lowering therapy in close relatives, physical signs suggestive of FH, and history of premature cardiovascular disease in family members. Close relatives included first-degree relatives younger than 50 years and second-degree relatives younger than 60 years. If at least one answer was positive, family history was considered positive, and the child was referred for further evaluation according to the Croatian National Program guidelines. During follow-up counseling sessions, parents were additionally encouraged to discuss family history information with other family members in order to improve reporting accuracy. During this process, additional family history information obtained from parents identified a positive family history of FH in three children who had initially been classified as FH-negative. These participants no longer met the inclusion criteria and were therefore excluded from the final analysis.

Waist circumference was measured by school physicians, while body height and body weight were measured by graduate nurses using calibrated standardized instruments. Children were measured without shoes and in light clothing. Body height was measured to the nearest 1 cm, body weight to the nearest 0.1 kg, and waist circumference to the nearest 1 mm.

Venous blood samples were collected into VACUETTE® tubes (catalog number 456089; Greiner Bio-One GmbH, Kremsmünster, Austria) containing a clot activator. Samples were centrifuged for 10 min at 3500 rpm at least 30 min after collection. TC levels were analyzed using the Beckman Coulter DxC 700 AU analyzer (Beckman Coulter, Inc., Brea, CA, USA) with the CDC (Abell–Kendall) CHO-POD method and Beckman Coulter reagents (catalog number OSR6116; Beckman Coulter, Inc., Brea, CA, USA). Fasting was not required for TC measurement [24]. TC was selected because it represents the primary biochemical parameter used within the Croatian National Program during routine school healthcare screening. Additional lipid parameters, including LDL-C, HDL-C, triglycerides, and non-HDL cholesterol, were not systematically available during the study period.

Inclusion criteria included all children from SDC, a Mediterranean region of Croatia, who attended systematic medical examinations between 1 April 2024, and 1 July 2024, had TC values between 5.1 mmol/L and 6.0 mmol/L and a negative family history of FH, and were assessed as healthy for regular school attendance. Children granted early enrollment into primary school following professional assessment were also eligible if they met the same criteria. Exclusion criteria included restricted diets due to food allergies and the presence of disabilities.

The study was conducted in School and Adolescent Medicine Clinics in SDC, a Mediterranean region of Croatia.

### 2.1. Statistical Analysis

Descriptive statistics were calculated for all study variables. Continuous variables are presented as medians, interquartile ranges (IQRs), minimum values, and maximum values. Normality of distribution was assessed using the Shapiro–Wilk test. As several variables demonstrated non-normal distribution (*p* < 0.05), and several behavioral variables derived from the Pre-PAQ questionnaire were ordinal in nature, non-parametric methods were considered the most appropriate and statistically conservative analytical approach for the available data. Between-group comparisons for total cholesterol (TC), anthropometric measures, dietary habits, physical activity, and sedentary behavior variables were performed using the Mann–Whitney U test at baseline and after the intervention period. In addition to the post-intervention between-group comparison, a descriptive change-from-baseline analysis was conducted for the primary outcome by subtracting baseline TC values from follow-up TC values. The mean change in TC was calculated for each group, and the between-group difference in mean change was reported descriptively. More complex baseline-adjusted treatment effect analyses, such as ANCOVA or repeated-measures/mixed-effects models, were not retained as the primary analytical approach due to the relatively small sample size, non-normal distribution of several variables, and ordinal characteristics of several questionnaire-derived outcomes.

The primary outcome of the study was post-intervention TC level compared between the intervention and control groups. Secondary outcomes included adherence to the Mediterranean diet, physical activity, and sedentary behavior variables assessed using the KIDMED and Pre-PAQ questionnaires.

For the primary outcome, the unadjusted mean difference at follow-up, approximate 95% confidence interval, and Cohen’s d effect size were calculated from group means and standard deviations to provide additional information on the magnitude and potential clinical relevance of the observed intervention effect.

An additional baseline-adjusted analysis for the primary outcome, with post-intervention total cholesterol as the dependent variable, study group as the main independent variable, and baseline total cholesterol as a covariate, was considered; however, due to the modest sample size and distributional characteristics of the available data, it was not retained as the main analytical approach.

Statistical significance was set at *p* < 0.05. Due to the exploratory nature of behavioral analyses and the relatively small sample size, analyses involving multiple physical activity and sedentary behavior variables were interpreted cautiously. The primary outcome, TC, was considered the confirmatory outcome, whereas physical activity and sedentary behavior variables were considered exploratory outcomes. No formal correction for multiple comparisons was applied because the behavioral analyses were considered exploratory and hypothesis-generating. Therefore, these findings should be interpreted with caution due to the potential risk of type I error. Data processing and statistical analyses were performed using JASP software (Version 0.20.0). Figures and tables were used to support visualization and interpretation of the results.

### 2.2. Ethical Approval

The study was conducted in accordance with the Declaration of Helsinki and approved by the Ethics Committee of the Teaching Institute of Public Health, Split-Dalmatia County (Class: 007-05-23-01/001; Registration number: 2181-103-01-23-10; approval date: 7 December 2023). Written informed consent was obtained from parents or legal guardians of all participants included in the study.

## 3. Results

During the 2024/2025 school year, a total of 4675 preschool children underwent systematic medical examinations in SDC, a Mediterranean region of Croatia. Parents of 587 children declined blood sampling for unknown reasons. Consequently, TC measurements were obtained for 4088 children.

Among the tested children, 3820 had TC values below 5.1 mmol/L and were excluded from further evaluation. The remaining 268 children had elevated TC values and were additionally assessed for family history of FH through telephone interviews with parents or legal guardians.

A positive family history of FH was identified in 165 children, while 16 children had TC values ≥ 6.1 mmol/L and were referred for further evaluation according to the Croatian National Program guidelines. The remaining 87 children had TC values between 5.1 mmol/L and 6.0 mmol/L with an initially negative family history of FH and were considered eligible for inclusion in the study.

Eligible participants were randomized using the Research Randomizer tool into either the control group (*n* = 44) or an intervention group (*n* = 43). During follow-up, parents of seven children withdrew from the study and declined further participation in counseling sessions and follow-up blood sampling. In addition, additional family history information obtained during follow-up identified a positive family history of FH in three children whose family history had initially been classified as negative. These participants no longer met the inclusion criteria and were therefore excluded from the final analysis.

Consequently, the final study sample consisted of 77 children, including 38 children in the control group and 39 children in the intervention group [Figure 2].

The main descriptive statistical parameters for the analyzed variables are presented in detail in Table 1. The table includes TC, body mass index (BMI), waist-to-height ratio (WHtR), and total KIDMED scores for both the intervention and control groups at baseline (M1) and after the intervention period (M2). Continuous variables are presented as medians and interquartile ranges (IQRs), together with minimum and maximum values, in order to provide a comprehensive overview of data distribution and variability within the study sample.

Assessment of normality using the Shapiro–Wilk test demonstrated that several variables deviated from a normal distribution. In addition, several behavioral variables derived from the Pre-PAQ questionnaire were ordinal in nature. Therefore, non-parametric statistical methods were considered the most appropriate and statistically conservative approach for further analyses.

### 3.1. Effect of the Intervention on Total Cholesterol (TC)

The Mann–Whitney U test was used to compare TC levels between the intervention and control groups at baseline (M1) and after the intervention period (M2) [Table 2]. This non-parametric test was selected because a normal distribution of variables could not be assumed.

At baseline, mean TC values were similar in both groups. The mean TC value in the intervention group was 5.341 mmol/L (SD = 0.226), while in the control group it was 5.332 mmol/L (SD = 0.222). No statistically significant difference between groups was observed at baseline (U = 759.5, *p* = 0.852), indicating comparability of the groups with respect to baseline TC levels.

At follow-up, mean TC values were lower than baseline values in the intervention group, decreasing from 5.341 mmol/L (SD = 0.226) to 4.977 mmol/L (SD = 0.414), while the control group showed a smaller decrease, from 5.332 mmol/L (SD = 0.222) to 5.137 mmol/L (SD = 0.410). Although the between-group difference at follow-up did not reach statistical significance (U = 574.0, *p* = 0.089), these findings suggest a favorable but non-significant trend toward lower TC values in the intervention group. The unadjusted mean difference in TC at follow-up was −0.160 mmol/L, with an approximate 95% confidence interval from −0.35 to 0.03, corresponding to a small-to-moderate effect size in favor of the intervention group (Cohen’s d = −0.39) [Table 3].

In addition to the post-intervention between-group comparison, a descriptive change-from-baseline analysis was conducted for the primary outcome [Table 4]. Mean TC decreased in both groups from baseline to follow-up. The reduction was greater in the intervention group than in the control group (−0.364 mmol/L vs. −0.195 mmol/L), resulting in a between-group difference in mean change of −0.169 mmol/L in favor of the intervention group. However, because the post-intervention between-group comparison did not reach statistical significance, this finding should be interpreted as a favorable trend rather than definitive evidence of intervention effectiveness.

**Figure 3:**
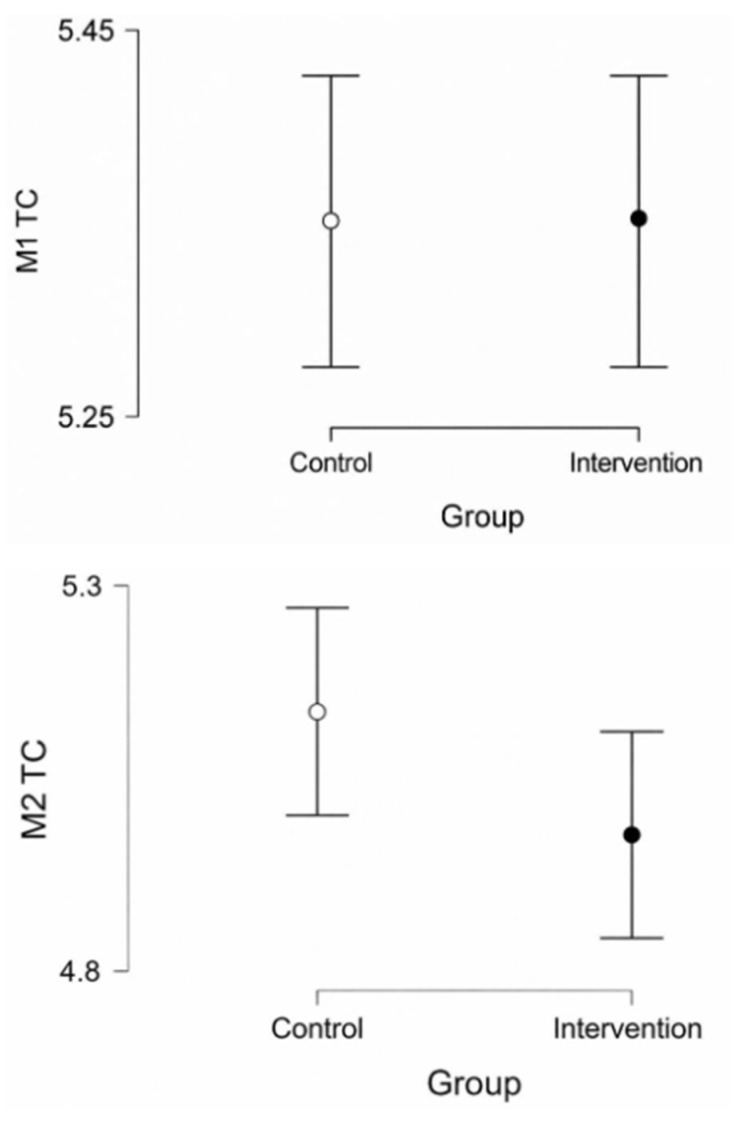
Total cholesterol (TC) values in the control and intervention groups at baseline (M1) and after intervention (M2).

Graphical representations [Figure 3] additionally illustrate differences in the direction of TC changes between groups during the intervention period.

Descriptively, increases in TC values appeared more frequent in the control group than in the intervention group; however, this observation was exploratory and was not formally tested statistically. Therefore, these findings should be interpreted cautiously.

### 3.2. Differences in BMI, WHtR, and KIDMED Before the Intervention

Baseline differences between the intervention and control groups in BMI, WHtR, and total KIDMED score were assessed using the Mann–Whitney U test [Table 5].

At baseline, the mean BMI value in the intervention group was 15.99 kg/m^2^ (SD = 2.42), compared with 16.75 kg/m^2^ (SD = 3.86) in the control group. No statistically significant difference between groups was observed (U = 753.0, *p* = 0.907).

WHtR values were similar in both groups. The intervention group had a mean WHtR value of 0.533 (SD = 0.035), while the control group also had a mean value of 0.533 (SD = 0.059), with no statistically significant difference between groups (U = 701.0, *p* = 0.687).

The total KIDMED score was slightly higher in the intervention group (M = 6.56, SD = 2.04) compared with the control group (M = 5.74, SD = 2.72); however, this difference was not statistically significant (U = 619.5, *p* = 0.214).

**Figure 4:**
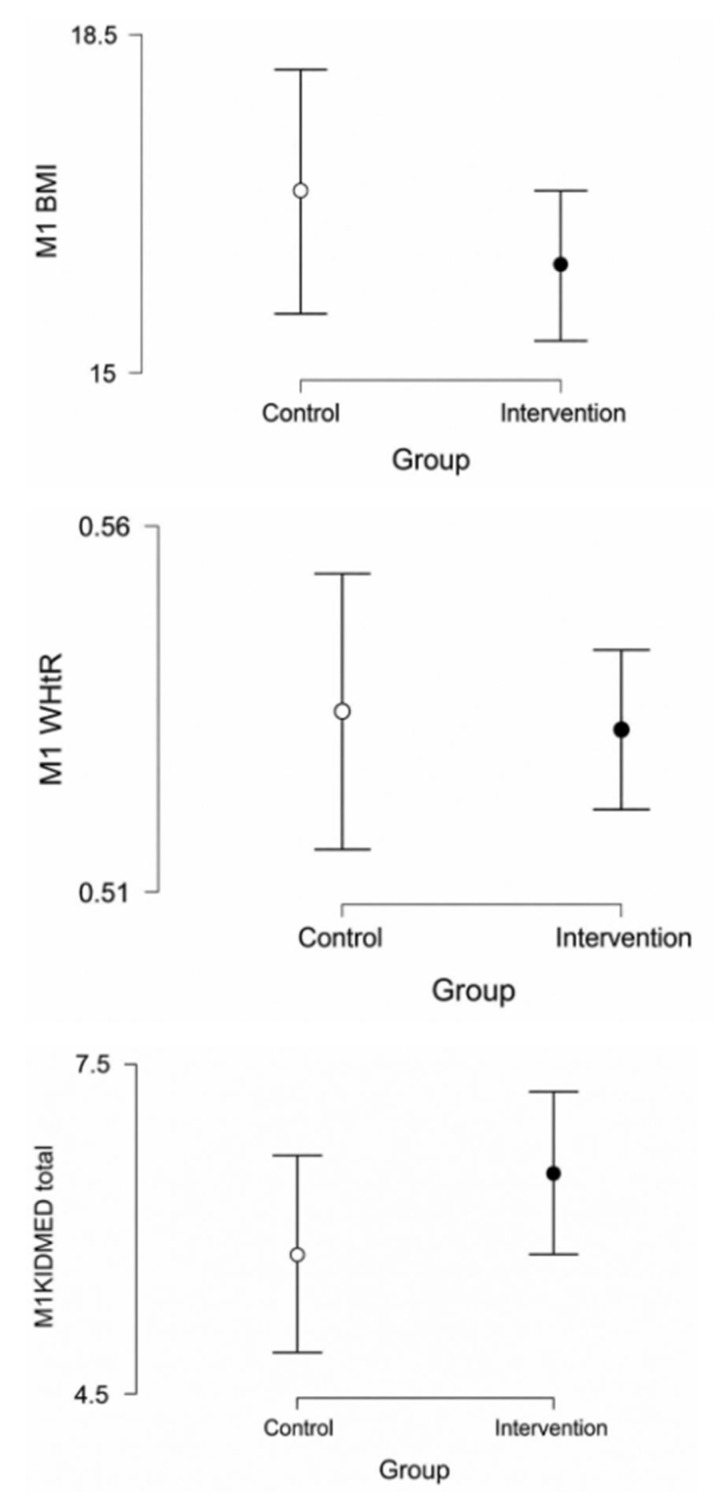
Body mass index (BMI), waist-to-height ratio (WHtR), and total KIDMED scores in the control and intervention groups at baseline (M1).

Overall, no statistically significant baseline differences between groups were observed for BMI, WHtR, or adherence to the Mediterranean diet, supporting the comparability of the groups before the intervention period [Figure 4].

### 3.3. Differences in BMI, WHtR, and KIDMED Scores After the Intervention

Differences between the intervention and control groups in BMI, WHtR, and total KIDMED score after the intervention period were assessed using the Mann–Whitney U test [Table 5].

After the intervention, the mean BMI value in the intervention group was 16.54 kg/m^2^ (SD = 2.61), while the control group had a slightly higher mean BMI value of 17.59 kg/m^2^ (SD = 4.52). However, the difference between groups was not statistically significant (U = 701.0, *p* = 0.687).

Similarly, WHtR values were lower in the intervention group (M = 0.455, SD = 0.043) compared with the control group (M = 0.479, SD = 0.065), although this difference did not reach statistical significance (U = 590.0, *p* = 0.125).

**Figure 5:**
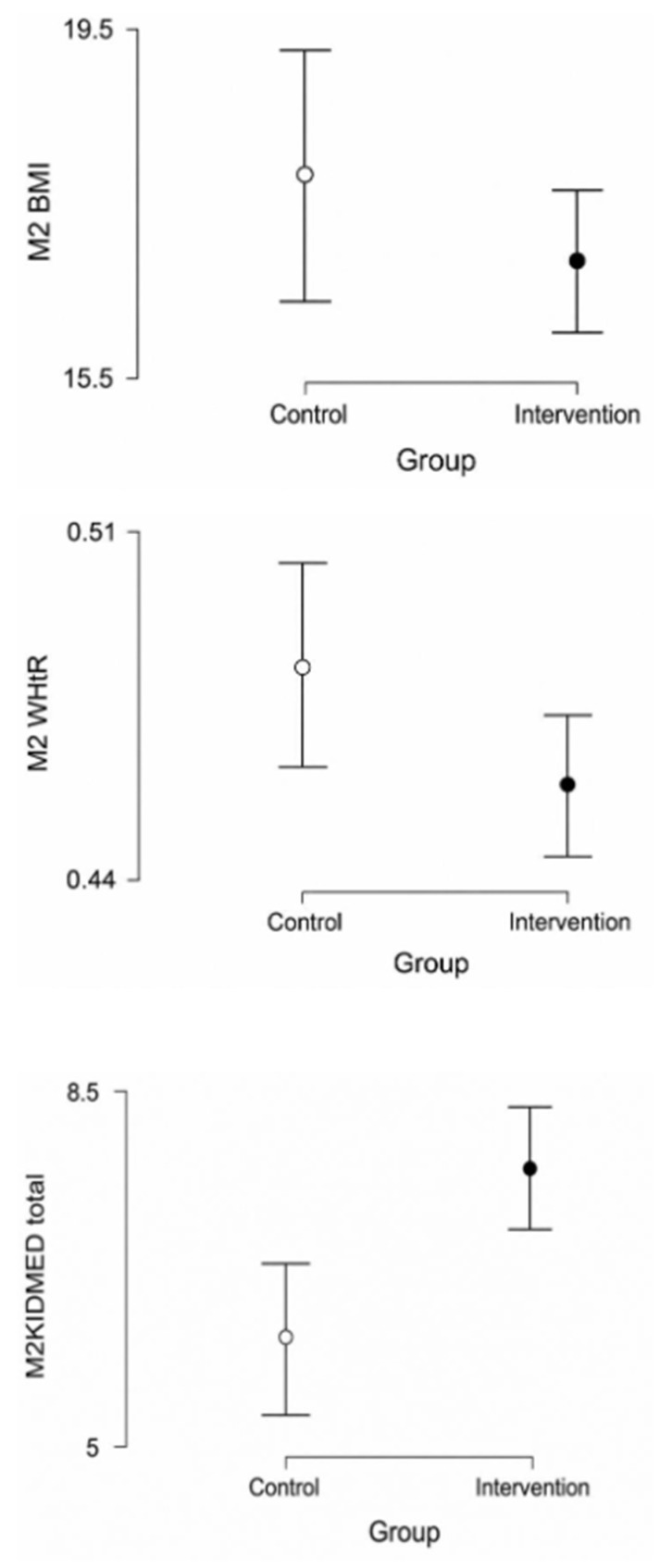
Body mass index (BMI), waist-to-height ratio (WHtR), and total KIDMED scores in the control and intervention groups after the intervention (M2).

In contrast, statistically significant differences were observed in total KIDMED scores after the intervention period. Children in the intervention group achieved higher KIDMED scores (M = 7.95, SD = 1.59) compared with children in the control group (M = 5.90, SD = 2.50). This difference was statistically significant (U = 1108.0, *p* < 0.001), suggesting improved adherence to the Mediterranean diet in the intervention group following the counseling intervention [Figure 5]. As adherence to the Mediterranean diet was a secondary outcome, this finding should be interpreted as supportive evidence of lifestyle change rather than as the primary confirmatory outcome of the study.

### 3.4. Physical Activity and Sedentary Behavior at Baseline (M1)

Baseline analyses of physical activity and sedentary behavior variables are presented in Table 6 and Table 7. Several statistically significant differences between the intervention and control groups were identified at baseline using the Mann–Whitney U test (*p* < 0.05), indicating that some behavioral variables were not fully balanced between groups prior to the intervention.

Children in the intervention group spent less time traveling by car on Sundays compared with children in the control group. In addition, parents perceived children in the intervention group as being more physically active compared with their peers.

Several sedentary behavior variables also differed between groups at baseline. Playing computer or video games on Sundays was reported less frequently in the intervention group compared with the control group. Similarly, time spent playing computer or video games on Sundays was lower in the intervention group.

Regarding physical activity behaviors, dancing on Sundays was reported more frequently in the intervention group compared with the control group, while time spent dancing was also higher in the intervention group.

These findings indicate the presence of several baseline differences in physical activity and sedentary behavior variables between groups prior to the intervention. Therefore, post-intervention behavioral findings should be interpreted cautiously. These baseline behavioral differences further support the interpretation of subsequent physical activity and sedentary behavior findings as exploratory rather than confirmatory.

### 3.5. Physical Activity and Sedentary Behavior After the Intervention (M2)

Post-intervention analyses of physical activity and sedentary behavior variables are presented in Table 8 and Table 9. Using the Mann–Whitney U test, statistically significant differences between the intervention and control groups were identified for several physical activity and sedentary behavior variables (*p* < 0.05).

Children in the intervention group showed higher levels of several physical activity variables after the intervention period. Weekly walking distance was higher in the intervention group compared with the control group. Participation in organized physical activity was also more frequent in the intervention group, while time spent in organized physical activity was higher than in the control group.

Variables related to vigorous physical activity also differed between groups. Running at a fast pace was reported more frequently in the intervention group compared with the control group. In addition, running at a fast pace on Sundays was more frequent in the intervention group.

Several sedentary behavior variables showed lower values in the intervention group after the intervention period. Playing computer or video games on the previous day was reported less frequently in the intervention group than in the control group, while time spent in these activities was also lower. Time spent watching television on Saturdays was lower in the intervention group compared with the control group. Similarly, playing computer games on Saturdays and time spent playing computer games on Saturdays were both lower in the intervention group. Playing computer games on Sundays was also reported less frequently in the intervention group.

Overall, these findings suggest higher levels of physical activity and lower levels of sedentary behavior in the intervention group after the intervention period. However, given the presence of several baseline differences between groups, the lack of adjustment for these baseline imbalances, and the large number of physical activity and sedentary behavior variables analyzed, these findings should be interpreted cautiously and considered exploratory rather than confirmatory.

## 4. Discussion

### 4.1. Effectiveness of the Counseling Intervention in Relation to TC Levels

This study evaluated the effects of a school physician-led counseling intervention on TC levels in children with elevated TC values and a negative family history of FH. After the one-year intervention period, children in the intervention group showed a favorable trend toward lower TC values compared with the control group. However, the between-group difference did not reach statistical significance and should therefore be interpreted cautiously.

The descriptive change-from-baseline analysis showed that mean TC decreased in both groups, with a greater reduction in the intervention group than in the control group. The unadjusted mean difference in post-intervention TC was −0.160 mmol/L, with a small-to-moderate effect size in favor of the intervention group (Cohen’s d = −0.39). Although the confidence interval included the null value and the between-group difference was not statistically significant, the observed reduction may still be clinically relevant in the context of childhood cardiovascular risk prevention. Even modest reductions in cholesterol levels during childhood may be meaningful if sustained over time, particularly among children already identified through screening as having elevated TC values. Therefore, these findings should be interpreted as preliminary and hypothesis-generating rather than confirmatory evidence of intervention effectiveness, and larger studies with longer follow-up are needed to determine whether such changes translate into sustained clinical benefit.

The findings of the present study are partially consistent with results from previous counseling interventions conducted in pediatric populations. A six-month study conducted in Italy investigated nutritional counseling among children aged 2–17 years with FH and reported improvements in both dietary habits and lipid profiles [25]. However, that study included children with confirmed FH, had a shorter intervention period, and did not evaluate physical activity outcomes. Another long-term study lasting seven years examined dietary interventions among children aged 8–10 years with hypercholesterolemia and reported reductions in LDL cholesterol together with improved dietary habits, although physical activity was not assessed [26].

In the present study, TC values decreased in both the intervention and control groups during follow-up. In the intervention group, mean TC values decreased to levels within the normal range (Mean 4.977, SD = 0.414), whereas TC values in the control group remained slightly elevated despite a modest decrease compared with baseline values (Mean 5.137, SD = 0.410). One possible explanation for improvements observed in both groups may be the baseline counseling and educational materials provided to all participants at the time elevated cholesterol levels were initially identified. This finding may suggest that even a single counseling session delivered by school physicians could support healthier lifestyle awareness in children and their families, although its independent effect on cholesterol-related outcomes cannot be determined based on the present study design and non-significant primary outcome.

### 4.2. Differences Between the Intervention and Control Groups in KIDMED, BMI, and WHtR

The results obtained using the KIDMED questionnaire demonstrated significantly higher scores in the intervention group after the intervention period (Mean 7.95, SD = 1.59) compared with the control group (Mean 5.90, SD = 2.50). These findings suggest improved adherence to the Mediterranean diet among children who received repeated counseling from school physicians. At baseline, KIDMED scores were relatively similar between groups (Mean 5.74–6.56), suggesting moderate adherence to the Mediterranean diet among children with hypercholesterolemia in SDC, Croatia.

The present findings are consistent with previous studies conducted in Mediterranean populations. A cross-sectional study performed in the same region reported poor or moderate adherence to the Mediterranean diet in 72% of 9-year-old children [27]. Similar declining trends in adherence to traditional Mediterranean dietary patterns among children and adolescents have also been described in Mediterranean countries such as Spain, Greece, and Italy [28]. Furthermore, a systematic review including 18 cross-sectional studies involving children and young adults aged 2–25 years demonstrated generally low adherence to the Mediterranean diet, with only a small proportion of participants showing high adherence [29]. Comparable findings were also reported in adults, with evidence suggesting a global decline in adherence to Mediterranean dietary patterns during the past decade [30].

Regarding anthropometric outcomes, children in the intervention group showed lower BMI values after the intervention (Mean 16.54, SD = 2.61) compared with the control group (Mean 17.59, SD = 4.52), although the difference was not statistically significant (U = 701.0, *p* = 0.687). Similarly, WHtR values were lower in the intervention group (Mean 0.455, SD = 0.043) compared with the control group (Mean 0.479, SD = 0.065), but this difference also did not reach statistical significance (U = 590.0, *p* = 0.125).

These findings should be interpreted cautiously because children in this age group undergo rapid growth and developmental changes over a relatively short period. The absence of statistically significant differences in BMI and WHtR may therefore reflect the limited duration of the intervention and the influence of normal growth patterns during childhood [31].

Similar findings have been reported in previous pediatric intervention studies. A one-year intervention study conducted in the United States among children aged 8–16 years reported decrease in body weight following lifestyle interventions focused on diet and physical activity [32]. Another study also demonstrated lower body weight after one year of intervention, although children remained within the obese category at follow-up [33].

### 4.3. Differences in Physical Activity and Sedentary Behavior

Previous intervention studies have demonstrated that lifestyle interventions focused on physical activity and dietary habits may contribute to improvements in cardiovascular risk profiles among children. The two-year PANIC study involving prepubertal children from the general population reported a small reduction in LDL cholesterol following combined dietary and physical activity counseling, although no significant effect on TC levels was observed [20]. Similarly, a meta-analysis evaluating aerobic exercise interventions among children and adolescents aged 5–19 years found no significant changes in TC levels, although favorable effects on triglycerides and HDL cholesterol were reported in children with overweight and obesity [34].

In the present study, children in the intervention group showed higher levels of several physical activity variables and lower levels of sedentary behavior following the counseling intervention. Post-intervention analyses indicated greater walking distance, increased participation in organized physical activities, and more time spent in physical activity among children in the intervention group. In addition, vigorous activities such as running at a fast pace, particularly during weekends, were reported more frequently in the intervention group. At the same time, several sedentary behaviors, including television viewing and computer or video game use during weekends, were reported less frequently in the intervention group.

These findings suggest that repeated counseling delivered by school physicians may contribute to healthier lifestyle behaviors in children with elevated cholesterol levels. However, interpretation of these findings should remain cautious because several physical activity and sedentary behavior variables differed between groups at baseline, and a large number of behavioral variables were analyzed without formal correction for multiple comparisons. This increases the possibility of type I error, meaning that some statistically significant findings may have occurred by chance. Therefore, individual significant results for physical activity and sedentary behavior should be interpreted as exploratory signals rather than definitive intervention effects.

The baseline differences observed for selected physical activity and sedentary behavior variables may have influenced the post-intervention findings. In particular, children in the intervention group already showed more favorable patterns for some behaviors at baseline, including lower computer or video game use and higher parent-perceived physical activity. These initial imbalances make it more difficult to attribute all post-intervention behavioral differences solely to the counseling intervention and may reduce the internal validity of conclusions regarding behavioral outcomes. Therefore, the physical activity and sedentary behavior results should be interpreted with particular caution, and future studies with larger samples and baseline-adjusted analyses are needed to provide more robust estimates of intervention effects on these outcomes.

The intervention group also demonstrated significantly higher KIDMED scores after the intervention period, indicating improved adherence to the Mediterranean diet. Improved dietary quality may reflect increased intake of vegetables, fruits, olive oil, and other components characteristic of Mediterranean dietary patterns. Similar findings have been reported in previous international studies. A long-term Finnish intervention study demonstrated beneficial effects of combined dietary and physical activity interventions on food choices and lifestyle behaviors [19]. In addition, a systematic review and meta-analysis reported associations between Mediterranean diet adherence and lower BMI, obesity prevalence, and WHtR in children and adolescents [35]. Previous studies have also shown that counseling interventions may increase participation in organized physical activity while reducing sedentary behaviors such as computer and video game use [36,37].

The present study demonstrated favorable trends in TC values together with improved adherence to the Mediterranean diet and favorable exploratory findings related to selected physical activity and sedentary behavior variables following the intervention. Nevertheless, because the between-group difference in TC levels did not reach statistical significance, conclusions regarding the effect of the intervention on TC levels should be interpreted cautiously. It is possible that improvements in dietary habits and physical activity were related to the observed favorable trends in TC values; however, causality cannot be inferred, and larger studies with longer follow-up periods and more comprehensive lipid assessments are needed to further clarify these relationships.

Although the observed changes in TC values were modest, even small improvements in cardiovascular risk factors during childhood may be relevant from a preventive public health perspective. However, the behavioral findings should be considered exploratory, and all findings should be interpreted within the context of the study’s methodological limitations.

### 4.4. Strengths and Limitations of the Study

A major strength of this study is the inclusion of all eligible children with the same health condition from a clearly defined Mediterranean region during a single screening period. This population-based approach increased the uniformity of the sample and reflects real-world conditions within the Croatian National Program. In addition, the randomized controlled design and the implementation of counseling interventions by school physicians within routine primary preventive healthcare represent important strengths of the study.

Nevertheless, several limitations should be acknowledged. First, the relatively small sample size may have limited the statistical power of the study, particularly regarding detection of statistically significant differences in TC levels between groups. In addition, an a priori sample size/power calculation was not performed. However, the sample represented the entire available population of children meeting the inclusion criteria within the observed geographic region during the study period, which limited the possibility of increasing the sample size.

Second, although an additional baseline-adjusted analysis for the primary outcome was considered and the analysis was complemented by a descriptive change-from-baseline assessment, more complex baseline-adjusted treatment effect analyses, such as ANCOVA or repeated-measures/mixed-effects models, were not retained as the primary analytical approach. The statistical analysis was primarily based on non-parametric between-group comparisons because of the relatively small sample size, non-normal distribution of several variables, and ordinal characteristics of several questionnaire-derived outcomes. Therefore, the findings should be interpreted cautiously and require confirmation in larger studies using pre-specified baseline-adjusted or repeated-measures analytical approaches.

Third, the study was not prospectively registered because it was designed as a public health research study based on a counseling intervention and questionnaire-based assessments rather than a clinical trial. Nevertheless, the study was approved by the appropriate Ethics Committee, and written informed consent was obtained from all parents or legal guardians of participating children.

Fourth, physical activity, sedentary behavior, and dietary habits were assessed using parent-reported questionnaires, introducing the possibility of recall bias and socially desirable responses. In addition, questionnaires were completed by either mothers or fathers, who may not have had equal insight into all aspects of the child’s daily activities and behaviors. An additional limitation relates specifically to the use of the Pre-PAQ questionnaire. Although the Pre-PAQ was originally developed for preschool-aged children, the children in this study were of preschool age at baseline but had entered primary school by the one-year follow-up assessment. The same questionnaire was retained at follow-up to ensure consistency and comparability between baseline and follow-up measurements. However, its use at follow-up may have reduced the precision of physical activity and sedentary behavior assessment in children who were no longer strictly within the preschool age range.

Fifth, several physical activity and sedentary behavior variables differed between groups at baseline despite randomization. Post-intervention analyses of these behavioral outcomes were not adjusted for baseline differences due to the relatively small sample size, non-normal distribution of variables, and ordinal characteristics of several Pre-PAQ variables. Although these outcomes were interpreted cautiously, residual baseline imbalance may have influenced some post-intervention findings. Furthermore, multiple behavioral variables were analyzed without formal correction for multiple comparisons, increasing the possibility of type I error. Therefore, physical activity and sedentary behavior findings should be considered exploratory and hypothesis-generating rather than confirmatory, and should be interpreted cautiously.

Sixth, only total cholesterol was systematically measured within the framework of the Croatian National Program. Additional lipid parameters, including LDL-C, HDL-C, triglycerides, and non-HDL cholesterol, were not routinely available, limiting the clinical interpretation of lipid-related outcomes.

Finally, the relatively short follow-up period may have limited the ability to detect more pronounced anthropometric changes, particularly in BMI and WHtR, during a period characterized by rapid growth and development in children.

Taken together, although the observed reduction in TC did not reach statistical significance, it may still be clinically relevant from a preventive public health perspective, particularly if modest improvements in cholesterol levels during childhood are sustained over time. However, these findings should be interpreted cautiously and considered preliminary and hypothesis-generating rather than confirmatory. Future research should confirm these results in larger multicenter studies with longer follow-up periods, greater statistical power, and pre-specified baseline-adjusted statistical approaches, such as ANCOVA or repeated-measures/mixed-effects models. In addition, the inclusion of comprehensive lipid profiles, including LDL-C, HDL-C, triglycerides, and non-HDL cholesterol, would allow a more precise evaluation of lipid-related cardiometabolic risk and the clinical implications of school physician-led counseling interventions.

## 5. Conclusions

The present study suggests that a school physician-led counseling intervention may be associated with improved adherence to the Mediterranean diet and favorable exploratory lifestyle-related changes among children with elevated TC levels. A favorable trend toward lower TC values was also observed in the intervention group, supported by descriptive change-from-baseline findings and a small-to-moderate effect size; however, the between-group difference did not reach statistical significance and should therefore be interpreted cautiously.

These findings suggest that repeated lifestyle counseling delivered within school healthcare settings may support healthier lifestyle awareness and selected health-related behaviors in children and could contribute to early cardiovascular disease prevention. However, because more complex baseline-adjusted or repeated-measures analyses were not retained as the primary analytical approach, several behavioral outcomes were exploratory, baseline imbalances were observed for selected physical activity and sedentary behavior variables, and no formal correction for multiple comparisons was applied, the findings should be interpreted cautiously. Further multicenter studies with larger sample sizes, longer follow-up periods, pre-specified confirmatory outcomes, and more comprehensive lipid assessments are needed to confirm these preliminary findings.

## Figures and Tables

**Table 1:** Descriptive statistics of total cholesterol (TC), body mass index (BMI), waist-to-height ratio (WHtR), and KIDMED scores in the intervention and control groups at baseline (M1) and after the intervention (M2).

		Median	IQR	Shapiro- Wilk	*p* Value	Minimum	Maximum
M1 TC	Intervention	5.300	0.400	0.892	0.001	5.100	5.900
M1 TC	Control	5.300	0.200	0.875	<0.001	5.100	6.000
M1 WHtR	Intervention	0.526	0.037	0.947	0.067	0.471	0.630
M1 WHtR	Control	0.525	0.055	0.942	0.049	0.430	0.701
M1 BMI	Intervention	15.609	2.288	0.838	<0.001	11.758	24.105
M1 BMI	Control	15.554	4.060	0.849	<0.001	12.160	29.238
M1KIDMED	Intervention	6.000	3.000	0.951	0.090	3.000	10.000
M1KIDMED	Control	6.000	4.000	0.962	0.220	0.000	11.000
M2 TC	Intervention	5.000	0.550	0.932	0.020	3.800	5.600
M2 TC	Control	5.150	0.575	0.971	0.408	4.200	5.900
M2 WHtR	Intervention	0.453	0.051	0.928	0.016	0.392	0.597
M2 WHtR	Control	0.462	0.081	0.908	0.004	0.368	0.682
M2 BMI	Intervention	15.984	2.249	0.859	<0.001	12.160	25.255
M2 BMI	Control	16.098	4.157	0.852	<0.001	12.303	31.565
M2KIDMED	Intervention	8.000	2.000	0.872	<0.001	4.000	10.000
M2KIDMED	Control	6.000	3.000	0.964	0.256	0.000	10.000

**Table 2:** Comparison of total cholesterol (TC) values between the intervention and control groups at baseline (M1) and after the intervention (M2) using the Mann–Whitney U test.

			U			*p*		
M1 TC			759.500			0.852		
M2 TC			574.000			0.089		
*Group Descriptives*								
	**Group**	**N**	**Mean**	**SD**	**SE**	**Coefficient of variation**	**Mean Rank**	**Sum Rank**
M1 TC	Intervention	39	5.341	0.226	0.036	0.042	39.474	1539.500
	Control	38	5.332	0.222	0.036	0.042	38.513	1463.500
M2 TC	Intervention	39	4.977	0.414	0.066	0.083	34.718	1354.000
	Control	38	5.137	0.410	0.066	0.080	43.395	1649.000

**Table 3:** Effect size and confidence interval for the primary outcome.

Primary Outcome	Intervention, Mean ± SD	Control, Mean ± SD	Mean Difference	95% CI	Cohen’s d	*p*-Value
M2 TC, mmol/L	4.977 ± 0.414	5.137 ± 0.410	−0.160	−0.35 to 0.03	−0.39	0.089

Note: Negative mean difference and Cohen’s d indicate lower total cholesterol values in the intervention group compared with the control group. CI, confidence interval; SD, standard deviation; TC, total cholesterol; M2, follow-up measurement.

**Table 4:** Descriptive change-from-baseline analysis of total cholesterol (TC).

Group	M1 TC Mean, mmol/L	M2 TC Mean, mmol/L	Mean Change, mmol/L
Intervention	5.341	4.977	−0.364
Control	5.332	5.137	−0.195
Between-group difference in mean change	-	-	−0.169

Note: Negative values indicate a reduction in TC from baseline (M1) to follow-up (M2). The between-group difference in mean change was in favor of the intervention group.

**Table 5:** Comparison of body mass index (BMI), waist-to-height ratio (WHtR), and total KIDMED scores between the intervention and control groups at baseline (M1) and after the intervention (M2) using the Mann–Whitney U test.

			U			*p*		
M1 BMI			753.000			0.907		
M1 WHtR			701.000			0.687		
M1 KIDMED total			619.500			0.214		
M2 BMI			701.000			0.687		
M2 WHtR			590.000			0.125		
M2 KIDMED total			1108.000			<0.001		
*Group Descriptives*								
	**Group**	**N**	**Mean**	**SD**	**SE**	**Coefficient of** **Variation**	**Mean Rank**	**Sum Rank**
M1 BMI	Control	38	16.746	3.857	0.626	0.230	39.316	1494.000
	Intervention	39	15.989	2.420	0.388	0.151	38.692	1509.000
M1 WHtR	Control	38	0.533	0.059	0.010	0.111	37.947	1442.000
	Intervention	39	0.533	0.035	0.006	0.065	40.026	1561.000
M1 KIDMED	Control	38	5.737	2.718	0.441	0.474	35.803	1360.500
	Intervention	39	6.564	2.036	0.326	0.310	42.115	1642.500
M2 BMI	Control	38	17.590	4.517	0.733	0.257	40.053	1522.000
	Intervention	39	16.538	2.605	0.417	0.158	37.974	1481.000
M2 WHtR	Control	38	0.479	0.065	0.011	0.136	42.974	1633.000
	Intervention	39	0.455	0.043	0.007	0.094	35.128	1370.000
M2 KIDMED	Control	38	5.895	2.502	0.406	0.424	29.342	1115.000
	Intervention	39	7.949	1.589	0.254	0.200	48.410	1888.000

**Table 6:** Mann–Whitney U test results for physical activity and sedentary behavior variables at baseline (M1).

Variable	U-Value	*p*-Value
M1PP9	446.000	0.002
M1PP17	933.500	0.032
M1PP99	583.000	0.022
M1PP100	590.000	0.030
M1PP115	909.000	0.027
M1PP116	908.500	0.030
M1PP9	time spent in the car last Sunday	
M1PP17	assessment of parents of activity compared to other children	
M1PP99	playing computer games or PlayStation on Sundays	
M1PP100	time spent playing computer games or PlayStation on Sundays	
M1PP115	dancing on Sundays	
M1PP116	time spent dancing on Sundays	

**Table 7:** Descriptive statistics for physical activity and sedentary behavior variables in the intervention and control groups at baseline (M1).

Variable	Group	N	Mean	SD	SE	Coefficient of Variation	Mean Rank
M1PP9	Intervention	39	0.209	0.409	0.066	1.953	31.436
	Control	38	0.472	0.508	0.082	1.078	46.763
M1PP17	Intervention	39	2.641	0.873	0.140	0.331	43.936
	Control	38	2.263	0.724	0.117	0.320	33.934
M1PP99	Intervention	39	1.103	0.307	0.049	0.279	34.949
	Control	38	1.316	0.471	0.076	0.358	43.158
M1PP100	Intervention	39	0.090	0.278	0.045	3.097	35.128
	Control	38	0.246	0.445	0.072	1.811	42.974
M1PP115	Intervention	39	1.385	0.493	0.079	0.356	43.308
	Control	38	1.158	0.370	0.060	0.319	34.579
M1PP116	Intervention	39	0.314	0.636	0.102	2.025	43.295
	Control	38	0.204	0.833	0.135	4.087	34.592

**Table 8:** Mann–Whitney U test results for physical activity and sedentary behavior variables after the intervention (M2).

Variable	U-Value	*p*-Value
M2PP12	981.000	0.013
M2PP19	899.500	0.028
M2PP25	926.000	0.023
M2PP36	564.500	0.006
M2PP37	559.500	0.005
M2PP41	542.500	0.039
M2PP47	946.000	0.024
M2PP66	532.000	0.028
M2PP69	525.000	0.002
M2PP70	532.000	0.003
M2PP99	579.000	0.031
M2PP107	939.500	0.012
M2PP12	how many meters did the child walk per week	
M2PP19	attending organized physical activity during the week	
M2PP25	time spent attending organized physical activity during the week	
M2PP36	playing computer games and PlayStation yesterday	
M2PP37	time spent playing computer games and PlayStation yesterday	
M2PP41	time spent walking at slow pace yesterday	
M2PP47	time spent running at fast pace yesterday	
M2PP66	time spent sitting or lying down watching TV on Saturdays	
M2PP69	playing computer games and PlayStation on Saturdays	
M2PP70	time spent playing computer games and PlayStation on Saturdays	
M2PP99	playing computer games and PlayStation on Sundays	
M2PP107	running at fast pace on Sundays	

**Table 9:** Descriptive statistics for physical activity and sedentary behavior variables between the intervention and control groups after the intervention (M2).

Variable	Group	N	Mean	SD	SE	Coefficient of Variation	Mean Rank	Sum Rank
M2PP12	Intervention	39	1915.385	2237.543	358.294	1.168	45.154	1761.000
	Control	38	988.158	1647.415	267.246	1.667	32.684	1242.000
M2PP19	Intervention	39	1.872	0.339	0.054	0.181	43.064	1679.500
	Control	38	1.658	0.481	0.078	0.290	34.829	1323.500
M2PP25	Intervention	39	1.122	1.357	0.217	1.210	43.744	1706.000
	Control	38	0.513	1.233	0.200	2.403	34.132	1297.000
M2PP36	Intervention	39	1.051	0.223	0.036	0.213	34.474	1344.500
	Control	38	1.289	0.460	0.075	0.356	43.645	1658.500
M2PP37	Intervention	39	0.026	0.112	0.018	4.357	34.346	1339.500
	Control	38	0.263	0.516	0.084	1.962	43.776	1663.500
M2PP41	Intervention	39	0.539	0.865	0.138	1.603	33.910	1322.500
	Control	38	0.792	0.977	0.158	1.234	44.224	1680.500
M2PP47	Intervention	39	0.417	0.674	0.108	1.616	44.256	1726.000
	Control	38	0.186	0.377	0.061	2.025	33.605	1277.000
M2PP66	Intervention	39	0.513	0.556	0.089	1.084	33.641	1312.000
	Control	38	0.928	0.825	0.134	0.889	44.500	1691.000
M2PP69	Intervention	39	1.077	0.270	0.043	0.251	33.462	1305.000
	Control	38	1.368	0.489	0.079	0.357	44.684	1698.000
M2PP70	Intervention	39	0.103	0.384	0.061	3.739	33.641	1312.000
	Control	38	0.401	0.706	0.115	1.759	44.500	1691.000
M2PP99	Intervention	39	1.154	0.366	0.059	0.317	34.846	1359.000
	Control	38	1.868	3.223	0.523	1.725	43.263	1644.000
M2PP107	Intervention	39	1.821	0.389	0.062	0.214	44.090	1719.500
	Control	38	1.553	0.504	0.082	0.325	33.776	1283.500

## Data Availability

The data presented in this study are available on request from the corresponding author. The data are not publicly available due to privacy and ethical restrictions related to research involving children and health-related data.

## Abbreviations

The following abbreviations are used in this manuscript:

BMI	Body Mass Index
CDC	Centers for Disease Control and Prevention
CHO-POD	Cholesterol Oxidase–Peroxidase
CI	Confidence Interval
CVD	Cardiovascular Disease
FH	Familial Hypercholesterolemia
HDL-C	High-Density Lipoprotein Cholesterol
IQR	Interquartile Range
KIDMED	Mediterranean Diet Quality Index for Children and Adolescents
LDL-C	Low-Density Lipoprotein Cholesterol
M1	Baseline Measurement
M2	Follow-Up Measurement
PANIC	Physical Activity and Nutrition in Children Study
Pre-PAQ	Preschool-Age Physical Activity Questionnaire
SD	Standard Deviation
SDC	Split-Dalmatia County
SE	Standard Error
TC	Total Cholesterol
WHO	World Health Organization
WHtR	Waist-to-Height Ratio
